# The role of interleukin-4 in acute kidney injury and chronic kidney disease: a literature review

**DOI:** 10.1186/s12882-026-04879-0

**Published:** 2026-03-05

**Authors:** Mengke Geng, Yuqian Guan, Keke Sun, Heng Jin

**Affiliations:** Department of Emergency Medicine, Tianjin Medical University General Hospital, Tianjin, 300052 China

**Keywords:** Interleukin-4 (IL-4), Macrophage polarization, AKI-to-CKD transition, Acute kidney injury (AKI), Chronic kidney disease (CKD), Macrophage-to-myofibroblast transition (MMT)

## Abstract

The transition from acute kidney injury (AKI) to chronic kidney disease (CKD) remains a major clinical challenge and contributes to substantial morbidity and mortality. Interleukin-4 (IL-4), a key cytokine in T helper 2 (Th2) immunity, appears to influence this trajectory in a context-dependent manner. In acute injury, IL-4 can limit inflammatory damage, support resolution, and facilitate tubular repair, in part through effects on macrophage phenotype. However, when IL-4 signaling persists during chronic injury, it may contribute to maladaptive remodeling, including activation of profibrotic myeloid and stromal programs and accumulation of extracellular matrix (ECM), thereby promoting renal fibrosis. This review summarizes evidence on IL-4’s biological properties, its canonical (Janus kinase [JAK]–signal transducer and activator of transcription 6 [STAT6]) and non-canonical (insulin receptor substrate [IRS]–phosphoinositide 3-kinase [PI3K]–protein kinase B [AKT]) signaling pathways, and its roles in renal diseases (including AKI, lupus nephritis, diabetic nephropathy, and other chronic glomerulopathies). We also evaluate the therapeutic rationale for targeting IL-4 signaling and highlight candidate molecular targets to mitigate renal fibrosis. Clarifying these determinants may help identify when and how IL-4–related pathways could be modulated to improve repair while limiting fibrosis.

## Introduction

The kidney is highly vulnerable to insults such as ischemia–reperfusion, nephrotoxins, and immune-mediated damage, making acute kidney injury (AKI) a frequent clinical problem [1]. Clinically, the AKI-to-chronic kidney disease (CKD) transition remains a major therapeutic challenge. Clinical data indicate that approximately 30% of AKI survivors fail to recover baseline function and progress to CKD [2, 3], with some ultimately progressing to end-stage renal disease (ESRD) requiring renal replacement therapy [4]. This transition is characterized by persistent tubular atrophy, vascular rarefaction, and progressive interstitial fibrosis [5].

Immune and stromal responses shape whether repair is adaptive or fibrotic. Interleukin-4 (IL-4), a canonical T helper 2 (Th2) cytokine, has been implicated in renal injury and repair [6]. IL-4 may be beneficial early after injury by promoting inflammation resolution and M2 macrophage–mediated repair [7]; however, sustained IL-4 signaling in chronic disease can promote fibrotic remodeling by stimulating fibroblast proliferation and extracellular matrix (ECM) production [8]. Mechanistically, IL-4 signals through specific receptor isoforms and activates downstream pathways, including Janus kinase (JAK)–signal transducer and activator of transcription 6 (STAT6) and insulin receptor substrate (IRS)–phosphoinositide 3-kinase (PI3K) signaling [9].

Here, we review the context-dependent functions of IL-4 in renal injury and repair, outlining its signaling mechanisms and examining whether targeting the IL-4 axis offers a viable strategy to uncouple reparative immunity from pathological fibrosis.

## Literature search methodology

We searched PubMed for English-language, peer-reviewed articles published from 2009 to 2025, using MeSH terms and keywords related to IL-4 and kidney disease. Our search strategy covered three main areas: (1) disease contexts (AKI, CKD, renal fibrosis, lupus nephritis, diabetic kidney disease, and selected chronic glomerular disorders); (2) mechanisms (macrophage polarization, macrophage-to-myofibroblast transition [MMT], single-cell RNA sequencing, and JAK/STAT6 signaling); and (3) therapeutic approaches (IL-4/IL-13-related interventions and JAK inhibitors). An example query was: (“Interleukin-4” OR IL-4) AND (AKI OR CKD OR “renal fibrosis”) AND (STAT6 OR JAK OR “macrophage polarization” OR MMT OR “single-cell RNA sequencing” OR scRNA-seq OR IL-13 OR “JAK inhibitors”). Priority was given to original studies providing mechanistic, experimental (in vitro/in vivo), or clinical evidence of IL-4 function in renal injury and repair, which were considered the primary inclusion criteria. Review articles were used to clarify pathway biology and to contextualize therapeutic targets; non-renal studies were cited only when necessary for mechanistic interpretation. Conference abstracts and editorials/letters were excluded. Relevant articles were identified by title and abstract screening, reviewed in full, and synthesized into mechanistic, clinical, and therapeutic sections.

## Biological characteristics

IL-4 is a defining cytokine of type 2 immunity, essential for orchestrating Th2 responses [10]. Structurally, IL-4 belongs to the short-chain cytokine superfamily and adopts the characteristic “up–up–down–down” four-helix bundle (α-helices A–D) with a small antiparallel β-sheet, stabilized by three disulfide bonds [6]. Human IL-4 is synthesized as a 153–amino acid pro-peptide and is proteolytically processed into a glycosylated 129–amino acid mature monomer (~ 17 kDa) [11]. IL-4 shows marked interspecies divergence: human and murine orthologs share ~ 43% sequence similarity. The murine protein lacks the prominent Helix C observed in primates, leading to species specificity and preventing human IL-4 from binding the murine receptor [12]. The *IL4* gene contains four exons and three introns and maps to chromosome 5q31.1 in humans (within the Th2 cytokine cluster) and chromosome 11 in mice [13, 14]. Transcription is regulated by GATA3, which promotes chromatin remodeling and enables binding of factors such as c-Maf and NFAT/AP-1 [11, 15]. Activated CD4⁺ T cells are the major source of IL-4; however, mast cells, basophils, and eosinophils can release IL-4 rapidly because they store pre-formed mature IL-4 in cytoplasmic granules, rather than relying on *de novo* synthesis after activation [12, 16–18].

## Associated signaling pathways

**Fig. 1:**
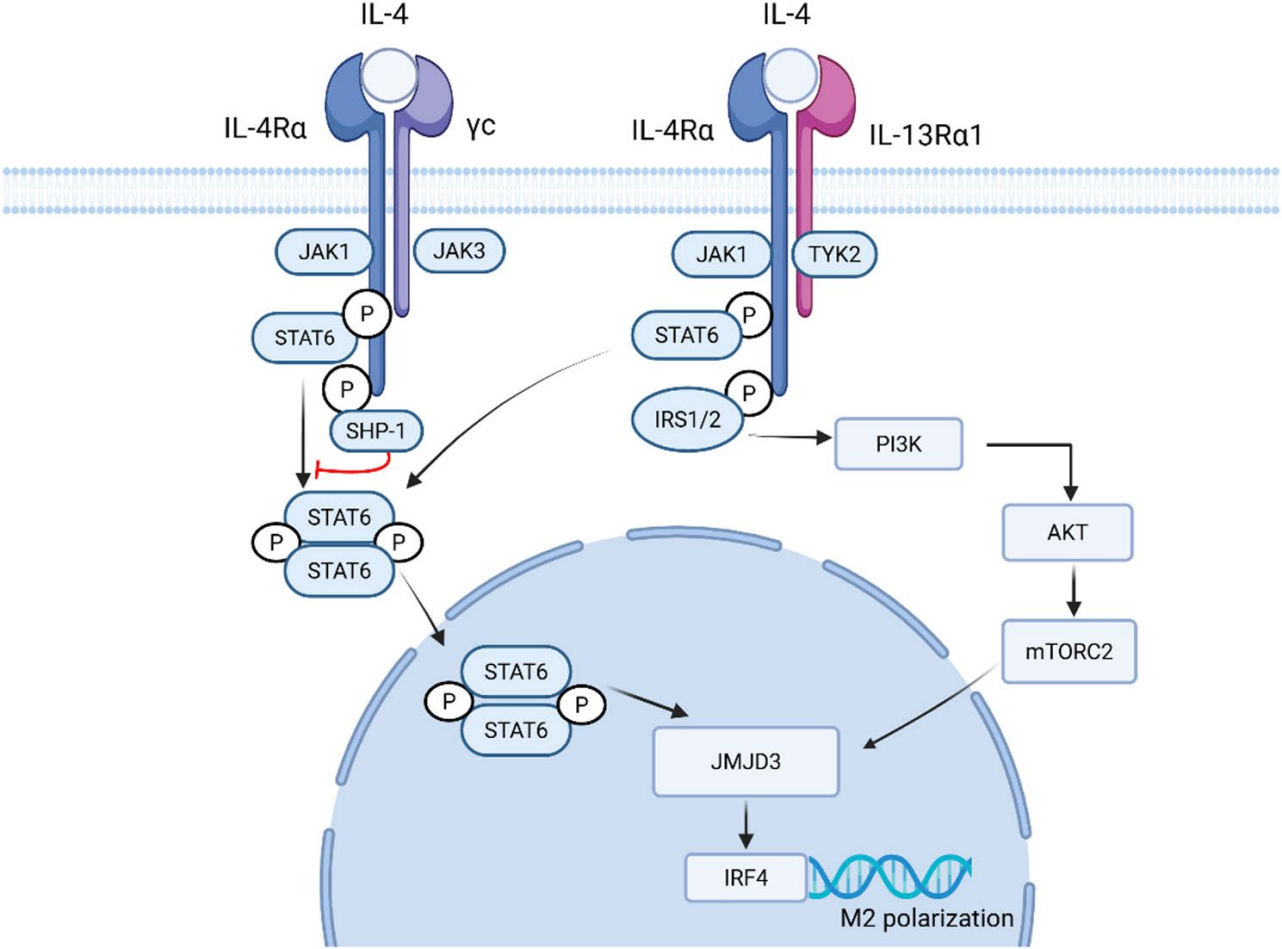
IL-4 Signaling Cascades in Renal Pathogenesis.IL-4 initiates signaling by binding to Type I (IL-4Rα/γc) or Type II (IL-4Rα/IL-13Rα1) receptor complexes, activating the associated Janus kinases (JAK1, JAK3, or TYK2). This leads to the phosphorylation, dimerization, and nuclear translocation of STAT6. In the nucleus, STAT6 induces the expression of the histone demethylase JMJD3, which epigenetically activates IRF4 to drive M2 macrophage polarization. Concurrently, IL-4 engages the non-canonical IRS1/2-PI3K-AKT-mTORC2 axis, which synergizes with the STAT6 pathway to regulate cellular metabolism and survival

IL-4 initiates signaling through two distinct dimeric receptor isoforms, both incorporating the IL-4 receptor α chain (IL-4Rα) (Fig. 1). Hematopoietic cells (e.g., T cells, mast cells) primarily utilize the Type I complex (IL-4Rα/common gamma chain [γc]), whereas non-hematopoietic tissues rely on the Type II complex (IL-4Rα/IL-13Rα1) [14, 19, 20]. Notably, in the renal context, tubular epithelial cells (TECs) express functional Type II receptors. Recent studies indicate that IL-4 signaling in these cells directly maintains epithelial homeostasis, for instance, by regulating the endocytic receptor Megalin via STAT6 activation [21]. Upon binding, IL-4 induces the transphosphorylation of receptor-associated Janus kinases (JAKs); specifically, IL-4Rα recruits JAK1, while the co-receptors recruit JAK3 (Type I) or JAK2/tyrosine kinase 2 (TYK2) (Type II), thereby establishing the active signaling pairs [22–25]. Activated JAKs phosphorylate tyrosine residues on the cytoplasmic domain of IL-4Rα. These phosphotyrosines serve as docking sites for STAT6, which subsequently homodimerizes and translocates to the nucleus to drive gene transcription [9].

Beyond the canonical cascade, IL-4 engages an alternative pathway via insulin receptor substrate (IRS1/2) adaptors. These proteins couple the receptor to the PI3K-protein kinase B (AKT)-mechanistic target of rapamycin complex 2 (mTORC2) axis, a signaling arm critical for metabolic reprogramming and M2 macrophage polarization [26]. Notably, this pathway acts synergistically with STAT6 to induce interferon regulatory factor 4 (IRF4), the master regulator of the M2 phenotype [27]. Full activation of the Irf4 program further requires the histone demethylase Jumonji domain-containing protein 3 (JMJD3). Mechanistically, JMJD3 removes repressive trimethylation of histone H3 lysine 27 [H3K27me3] marks at the Irf4 promoter, thereby opening the chromatin landscape to facilitate robust gene expression [28].

Finally, signaling is negatively regulated via an immunoreceptor tyrosine-based inhibitory motif (ITIM) on IL-4Rα. This motif recruits the phosphatase Src homology region 2 domain-containing phosphatase-1 (SHP-1), which dampens STAT6 signaling by dephosphorylating key intermediates [29].

## Context-dependent roles of IL-4 in renal pathology

Renal injury often progresses through a continuum from AKI to acute kidney disease (AKD) and CKD, imposing a significant burden on global health [30, 31]. Evidence from experimental and clinical studies implicates IL-4 as an important regulator across this spectrum. Table 1 summarizes reported IL-4–related phenotypes in murine kidney disease models. Below, we examine the specific mechanisms driven by IL-4 in AKI, lupus nephritis (LN), diabetic nephropathy (DN), and other chronic glomerulopathies.

**Table 1:** Mechanistic effects of IL-4 in kidney disease models

Study	Years	Kidney injury model	Main goal of the study	Main findings	References
Zhang et al.	2017	AKI (IRI & DT-induced)	To investigate whether IL-4/IL-13 signaling is required for the switch from pro-inflammatory to reparative M2a macrophages during the recovery phase of acute injury.	Confirmed that the IL-4/STAT6 axis is indispensable for epithelial recovery in both ischemic and toxic models; deficiency leads to failure of M2a polarization, delayed structural regeneration, and exacerbated secondary fibrosis.	[7]
Barrera-Chimal et al.	2018	AKI (IRI)	To elucidate the mechanism by which myeloid mineralocorticoid receptor (MR) activation represses IL-4 receptor signaling and promotes maladaptive repair.	Revealed that myeloid MR downregulates IL-4R expression; MR antagonism (Finerenone) restores IL-4 responsiveness, potentiating M2 polarization and preventing the transition to chronic fibrosis.	[32]
Mao et al.	2020	AKI (IRI)	To evaluate the feasibility and efficacy of adoptive transfer of ex vivo IL-4-polarized M2 macrophages as a cell-based therapy for repairing renal injury.	Validated that adoptive transfer of peritoneal M2 macrophages directly promotes tubular epithelial cell proliferation and attenuates inflammation via IL-4-mediated mechanisms.	[33]
Zhou et al.	2024	AKI (IRI)	To determine if the integrin CD11b acts as an upstream negative regulator of the IL-4/STAT6 signaling axis in the context of AKI-to-CKD progression.	Identified CD11b as a physiological “brake”; its deficiency inactivates Lyn kinase, thereby unleashing spontaneous IL-4/STAT6 activation and enhancing protective M2 polarization.	[34]
Peruchetti et al.	2020	Protein Overload (BSA-induced)	To examine the protective role of IL-4Rα in maintaining tubular epithelial homeostasis and endocytic function under protein overload stress.	Demonstrated that IL-4 maintains the expression of the endocytic receptor Megalin via STAT6 activation; deficiency exacerbates tubulointerstitial inflammation and proteinuria.	[21]
Liang et al.	2017	Toxic Injury (FA) & UUO	To dissect the specific contribution of IL-4Rα signaling in bone marrow-derived cells versus intrinsic renal cells to myofibroblast accumulation and fibrosis.	Established that IL-4Rα specifically on bone marrow-derived cells acts as the primary driver of fibroblast accumulation; genetic ablation prevents the transition of monocytes into collagen-producing fibrocytes.	[35]
Jiao et al.	2021	Toxic Injury (FA)	To examine whether STAT6 is the critical downstream effector mediating IL-4-induced myeloid fibroblast activation and ECM production	Demonstrated that genetic deletion of STAT6 significantly reduces the accumulation of myeloid-derived fibroblasts and blocks the production of collagen and fibronectin.	[36]
Chen et al.	2021	Toxic Injury (FA)	To investigate the role of the transcription factor IRF4 as a molecular switch in regulating inflammation and the fibrotic phenotype of macrophages.	Identified IRF4 as the terminal transcriptional checkpoint; its deficiency suppresses pro-inflammatory cytokines and abolishes the transition of macrophages into myofibroblasts.	[37]
Liu et al.	2021	Toxic Injury (FA) & UUO	To identify the specific immune cell subset responsible for producing pro-fibrotic IL-4 and to determine the role of NKT cells in driving MMT.	Identified Natural Killer T (NKT) cells as a critical source of IL-4; CD1d-mediated NKT activation fuels the MMT and exacerbates fibrosis.	[8]
Liang et al.	2022	Toxic Injury (FA) & UUO	To explore the epigenetic mechanism by which the JMJD3/IRF4 axis downstream of IL-4 mediates MMT in renal fibrosis.	Elucidated that IL-4 induces JMJD3 to epigenetically activate IRF4; genetic or pharmacological inhibition of this axis blocks MMT and attenuates maladaptive remodeling.	[38]
Zhou et al.	2024	Obstructive Injury (UUO)	To assess whether the anti-rheumatic drug Iguratimod attenuates renal fibrosis by targeting the IL-4/STAT6/Src signaling axis.	Showed that Iguratimod dampens the IL-4/STAT6 pathway and directly inhibits Src kinase activation, thereby blocking MMT and reducing macrophage infiltration.	[39]
Chen (Y.) et al.	2025	Obstructive Injury (UUO)	To investigate the role of the Kv1.3 potassium channel in regulating mitochondrial membrane potential during M2 polarization.	Revealed that Kv1.3 maintains mitochondrial potential for STAT6 signaling; blocking Kv1.3 disrupts metabolic fitness and significantly ameliorates fibrosis.	[40]
Liang (W.) et al.	2025	Obstructive Injury (UUO)	To explore the potential of the gut metabolite Sodium Butyrate (NaB) to inhibit M2 polarization via the LKB1 kinase pathway.	Discovered that NaB activates LKB1, which acts as a novel negative regulator to suppress IL-4/STAT6 signaling and downstream fibroblast activation.	[41]
Liang (C.L.) et al.	2021	LN(Pristane-induced)	To elucidate the immunomodulatory mechanism of Paeoniflorin (TGP) in lupus nephritis	Demonstrated that Paeoniflorin (TGP) activates the IL-4/STAT6/PD-L2 axis, enhancing immune tolerance and ameliorating nephritis.	[42]
Tchen et al.	2024	LN(Pristane-induced Lupus; Lyn-/- Spontaneous Lupus)	To investigate the mechanism by which basophils promote T follicular helper (Tfh) cell accumulation and autoantibody production in SLE.	Basophil-derived IL-4, synergizing with PD-L1, drives pathogenic Tfh2 cell accumulation and autoreactive IgG production, thereby exacerbating lupus nephritis.	[43]
Gu et al.	2024	DN(*db/db* mice)	To evaluate the therapeutic efficacy of systemic M2 macrophage infusion in preventing diabetic glomerulopathy	Showed that IL-4-primed M2 macrophages suppress meta-inflammation and fibrosis by downregulating the JAK2/STAT3 signaling pathway	[44]
Kim et al.	2017	MCD / Podocytopathy (IL-4 Overexpression; Targeted B-cell Activation)	To test whether B cell-derived IL-4 can directly induce podocyte injury and proteinuria independent of antibodies.	IL-4 directly targets podocytes via STAT6 to induce foot process effacement and proteinuria; JAK inhibition ameliorates this injury.	[45]
Cavalcante et al.	2019	Hypertensive Nephropathy (Transgenic TGM(rAOGEN)123 mice)	To determine the polarization status of macrophages and the role of IL-4-induced M2 phenotype in hypertensive kidney damage.	In hypertension, renal macrophages are predisposed to M2 polarization upon IL-4/IL-13 stimulation, secreting pro-fibrotic TGF-β1 and YM1, thus contributing to renal fibrosis.	[46]
Yao et al.	2024	MN(C-BSA induced)	To investigate the role of miR-223 in mediating the therapeutic effects of cyclophosphamide (CTX) on MN and macrophage polarization.	CTX and miR-223 overexpression promote M2 macrophage polarization, increase anti-inflammatory cytokines (IL-4, IL-13), and alleviate renal injury in MN.	[47]

Abbreviations: AKI, acute kidney injury; BSA, bovine serum albumin; CKD, chronic kidney disease; CTX, cyclophosphamide; DN, diabetic nephropathy; DT, diphtheria toxin; ECM, extracellular matrix; FA, folic acid; IgG, immunoglobulin G; IL-4, interleukin-4; IL-13, interleukin-13; IL-4Rα, interleukin-4 receptor alpha; IRI, ischemia–reperfusion injury; JAK, Janus kinase; JAK2, Janus kinase 2; LN, lupus nephritis; LKB1 (STK11), liver kinase B1 (serine/threonine kinase 11); MCD, minimal change disease; miR-223, microRNA-223; MMT, macrophage-to-myofibroblast transition; MN, membranous nephropathy; MR, mineralocorticoid receptor; NaB, sodium butyrate; NKT, natural killer T; PD-L1, programmed death-ligand 1; PD-L2, programmed death-ligand 2; SLE, systemic lupus erythematosus; SRC/Src, SRC proto-oncogene, non-receptor tyrosine kinase; STAT3, signal transducer and activator of transcription 3; STAT6, signal transducer and activator of transcription 6; Tfh, T follicular helper; TGF-β1, transforming growth factor beta 1; TGP, total glucosides of paeony; UUO, unilateral ureteral obstruction; Kv1.3, voltage-gated potassium channel Kv1.3

### AKI and maladaptive repair

**Fig. 2:**
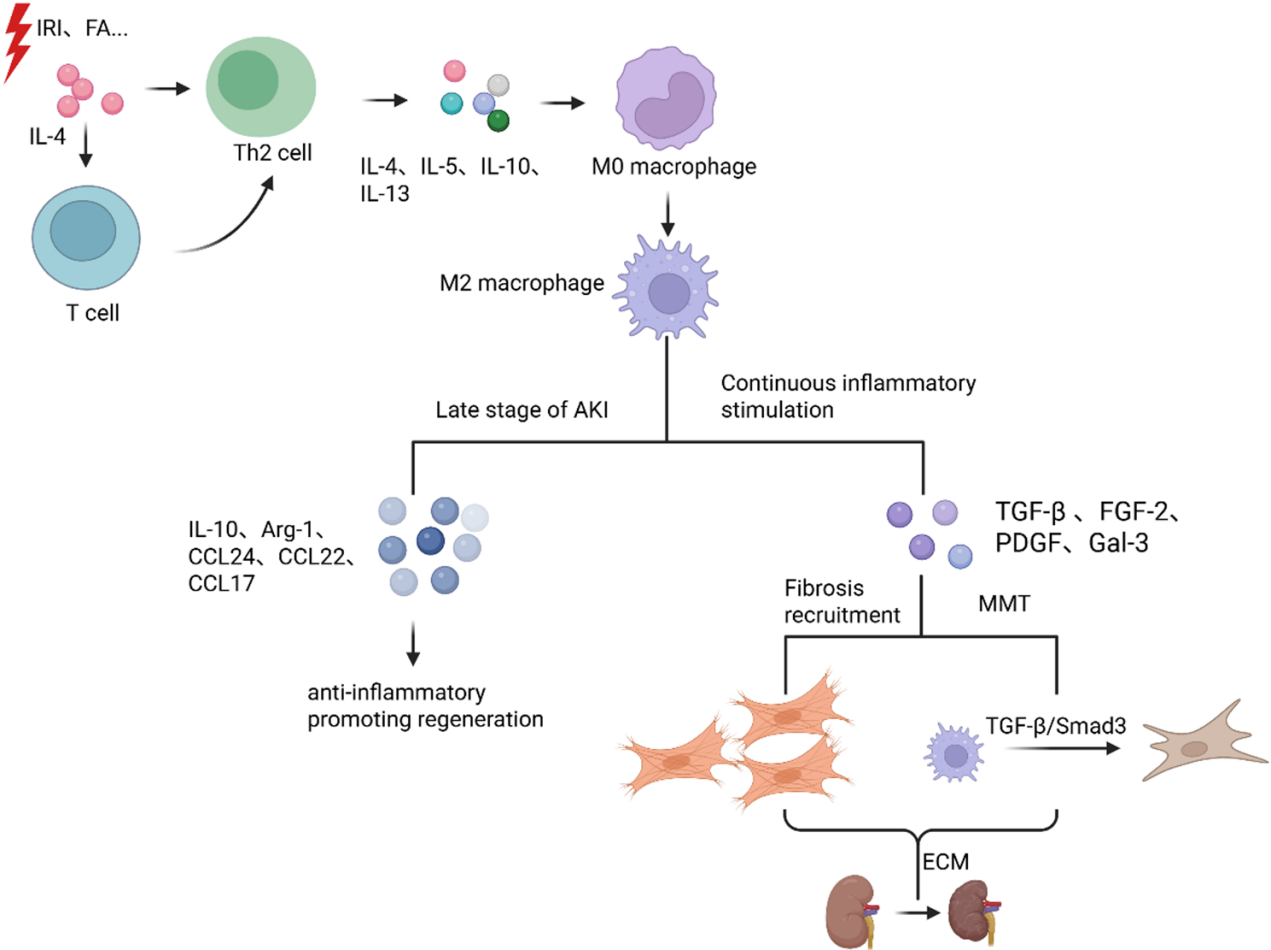
Divergent macrophage trajectories driven by IL-4 in renal pathology. IL-4 initially promotes Th2 differentiation and M2 polarization to facilitate tissue repair via anti-inflammatory mediators. However, persistent inflammatory stimuli trigger a maladaptive switch: M2 macrophages secrete profibrotic cytokines (TGF-β, PDGF) and undergo MMT, thereby driving ECM accumulation and fibrosis

AKI encompasses a diverse range of syndromes triggered by ischemia–reperfusion injury (IRI), nephrotoxins, or urinary tract obstruction (frequently modeled by unilateral ureteral obstruction, UUO). Despite distinct initiating insults, the progression to chronic disease is often driven by incomplete repair, leading to maladaptive remodeling and fibrosis [48–50]. Across these settings, IL-4 shows context-dependent effects. Mechanistic studies typically rely on complementary murine models: IRI and toxin-induced injury are used to interrogate acute inflammation and early repair, whereas UUO models sustained mechanical stress and progressive fibrogenesis. Figure 2 summarizes this shift from early anti-inflammatory or reparative responses to later profibrotic programs. The following subsections describe IL-4 functions in each context.

#### IRI

IRI, commonly associated with cardiac surgery or kidney transplantation, is a leading driver of AKI. The initial metabolic compromise during ischemia is followed by reperfusion, which triggers a robust inflammatory cascade [51, 52]. Murine IRI models have been instrumental in defining IL-4–dependent mechanisms during post-ischemic repair. Loss-of-function studies provide key evidence for a protective role of the IL-4 axis. Zhang et al. [7] reported that *Il4/Il13-deficient* mice developed more severe renal dysfunction and accelerated fibrosis than wild-type controls. This phenotype was associated with impaired macrophage polarization: *Il4-deficient* mice maintained a pro-inflammatory M1-like state and failed to adopt a reparative M2a program, supporting a role for IL-4–JAK3–STAT6 signaling in recovery after ischemic injury. Subsequent work has examined upstream regulators that restrain IL-4 signaling. Zhou et al. [34] identified integrin CD11b as a physiological brake; CD11b deficiency reduced Lyn activity, enhanced IL-4/STAT6 signaling, and was associated with protection from fibrosis in a murine setting. Barrera-Chimal et al. [32] further showed that myeloid mineralocorticoid receptor (MR) signaling suppresses IL-4 receptor expression, and that MR antagonism with finerenone increased IL-4 responsiveness, promoted M2 polarization, and attenuated AKI-to-CKD transition in murine and porcine models. In addition, cell-based approaches have been explored. Mao et al. [33] reported that adoptive transfer of ex vivo IL-4–polarized M2 macrophages into the renal cortex promoted tubular epithelial proliferation and reduced injury in mice. Collectively, these findings support a consistent theme in IRI models: intact IL-4 signaling facilitates repair, and therapeutic strategies that enhance this pathway—either by relieving inhibitory checkpoints (e.g., CD11b/MR-related restraints) or by providing IL-4–programmed effector cells—may mitigate post-ischemic CKD progression.

#### Toxin-induced renal injury

The kidney’s unique role in concentrating and excreting metabolites renders it highly vulnerable to nephrotoxicity. Subsequent drug or toxin exposures frequently result in prominent tubular injury [53], a pathology commonly modeled using folic acid (FA), cisplatin, aminoglycosides, or diphtheria toxin (DT) [54]. In these settings, IL-4 has been linked to tubular injury responses and repair [55].

##### Protective role in acute toxic injury

In models with reversible tubular injury, IL-4 signaling is generally associated with repair. Using a DT-induced proximal tubule injury model, Zhang et al. [7] showed that IL-4–JAK3–STAT6 signaling was required for epithelial recovery. Disruption of the pathway—via genetic deletion of *Il4/Il13* or pharmacologic JAK3 inhibition (tofacitinib)—blocked the macrophage shift toward a reparative M2a program and was associated with delayed regeneration and secondary fibrosis. Peruchetti et al. [21] examined albumin overload–induced tubular injury and reported that IL-4 maintained expression of the endocytic receptor megalin in proximal tubule cells through STAT6 activation; *Il4rα* deficiency impaired this response and correlated with worsened tubulointerstitial inflammation and proteinuria. Together, these studies highlight a protective role for IL-4 in acute toxic injury through both immune polarization and direct effects on tubular epithelial function.

##### The IL-4 axis as a driver of maladaptive transition

High-dose FA administration is widely used to model the progression from acute tubular necrosis to robust interstitial scarring [56]. In this setting, sustained IL-4 signaling has been implicated in maladaptive remodeling.

Rodent studies further suggest a defined signaling sequence underlying this shift, beginning with the cellular sources of IL-4 and extending to downstream profibrotic pathways. Liu et al. [8] identified natural killer T (NKT) cells as a major source of IL-4 in FA nephropathy, with injury-associated CD1d signaling promoting NKT activation and IL-4 secretion. Liang et al. [35] used bone marrow transplantation to show that IL-4Rα expression in bone marrow–derived cells was required for the profibrotic response; restricting IL-4Rα deficiency to myeloid compartments reduced myofibroblast accumulation and interstitial fibrosis. Downstream, Jiao et al. [36] reported that STAT6 was necessary for expansion of CD45⁺/platelet-derived growth factor receptor-β[PDGFR-β] fibroblast-like populations and for M2 polarization, with parallel reductions in collagen I and fibronectin deposition when *Stat6* was ablated.

Epigenetic mechanisms appear to reinforce this program. Liang et al. [38] reported that IL-4/STAT6 signaling increased JMJD3, which removes repressive H3K27me3 marks and increases chromatin accessibility at the *Irf4* promoter. Chen et al. [37] identified IRF4 as a downstream transcriptional checkpoint; *Irf4* deficiency reduced expression of inflammatory mediators (e.g., IL-6 and TGF-β1) and markedly diminished α-SMA induction in injured kidneys. Together, these studies outline a pathway in which an NKT–IL-4–IL-4Rα–STAT6 axis engages JMJD3/IRF4 to promote MMT, generating CD206⁺/alpha-smooth muscle actin[α-SMA] collagen-producing cells and amplifying fibrosis.

##### Synthesis and conclusion

Across toxin-induced AKI models, IL-4 exhibits a temporal split. During early, reversible tubular injury, IL-4–dependent programs support resolution and repair. Under persistent or severe injury (e.g., high-dose FA), sustained IL-4 signaling becomes profibrotic and promotes maladaptive transition. Importantly, these conclusions are context- and cell-type–dependent. As discussed later (Sect. “Other chronic nephropathies”), IL-4 can also act directly on podocytes, where exposure has been linked to acute foot process effacement and proteinuria [45].

#### UUO

UUO is a standard model of tubulointerstitial fibrosis driven by persistent mechanical stress [57]. Unlike transient injury models, UUO reduces the likelihood of a dominant early reparative phase and favors sustained inflammatory and fibrogenic signaling.

Mirroring findings in FA nephropathy, studies in UUO implicate the NKT–IL-4–IL-4Rα–STAT6–JMJD3–IRF4 axis in myeloid reprogramming and MMT. Liang et al. [35, 38] reported that this cascade drives epigenetic changes in bone marrow–derived cells and their transition toward myofibroblast-like phenotypes. Liu et al. [8] similarly reported that NKT cells are a major IL-4 source under mechanical stress and that CD1d-dependent activation contributes to MMT.

Pharmacologic studies have proposed additional regulatory nodes. Zhou et al. [39] reported that iguratimod treatment in UUO mice reduced MMT and fibrosis and suggested a role for Src signaling in coupling IL-4 pathways to fibroblast differentiation. Chen (Y.) et al. [40] used the Kv1.3 blocker PAP-1 to implicate a metabolic requirement for effective STAT6 signaling, linking Kv1.3-dependent mitochondrial membrane potential to profibrotic macrophage responses. Liang (W.) et al. [41] proposed LKB1 as a negative regulator; upregulating LKB1 reduced STAT6 phosphorylation and limited M2 polarization. Overall, murine UUO studies place IL-4 as a profibrotic driver under sustained stress and nominate upstream regulators (e.g., Src, Kv1.3, LKB1) as potential intervention points. Given the heterogeneity of human obstructive and fibrotic kidney disease, validating these axes in human tissue across the AKI–AKD–CKD continuum remains an important next step.

### CKD

CKD arises from diverse etiologies, including primary glomerulonephritis, hypertensive nephrosclerosis, and secondary autoimmune or metabolic disorders [58]. Genetic association studies also implicate the IL-4 axis. Arababadi et al. [59] found significant differences in genotype distributions (C/C, T/C, T/T) at the *IL-4* gene promoter − 590 region between nephropathic patients and healthy controls, suggesting a link to disease susceptibility. This complements earlier findings by Mai et al. [60] on other susceptibility loci (e.g., rs1800469, rs1800470). Mechanistically, it is hypothesized that these genetic variations may influence disease outcomes by modulating IL-4 transcriptional efficiency, potentially altering the individual’s capacity for M2 macrophage polarization—a key factor in the balance between renal repair and fibrosis.

#### LN

Systemic lupus erythematosus (SLE) is frequently complicated by LN, driven by immune complex deposition and dysregulated innate and adaptive immunity [61]. Active disease is often characterized by Th1/Th17-skewed inflammation and tissue injury [62, 63]. Peng et al. [64] reviewed evidence that active SLE is associated with predominance of M1-like macrophage signatures, whereas remission correlates with a shift toward M2-associated profiles. In this context, IL-4 may counterbalance Th1-biased inflammation and contribute to immune regulation.

Using a pristane-induced LN model, Liang (C.L.) et al. [42] reported that paeoniflorin (TGP) ameliorated renal injury and was associated with activation of IL-4/STAT6 signaling and increased programmed death-ligand2(PD-L2) expression on macrophages. PD-L2 can inhibit T-cell activation; the authors proposed that IL-4/STAT6–driven PD-L2⁺ macrophage polarization attenuated pathogenic responses, reduced anti-dsDNA levels, and slowed chronic progression. However, type 2 immunity may also promote pathology in LN. Tchen et al. [43] identified basophils as an IL-4 source in secondary lymphoid organs in lupus-prone mice and linked basophil-derived IL-4 to expansion of T follicular helper 2 (Tfh2) cells. These Tfh2 cells promoted IgE class switching, and IgE deposition in kidneys has been associated with active nephritis [65]. In addition, Th2 cells may contribute to glomerulosclerosis or recruit eosinophils via IL-5 [43]. Thus, IL-4 appears to play a dual role in LN, with the net outcome determined by the interplay between regulatory PD-L2–associated pathways and pathogenic Tfh2/IgE-dependent mechanisms.

#### DN

DN is a major microvascular complication of diabetes and a leading cause of ESRD globally [66]. Its pathogenesis involves chronic low-grade “meta-inflammation,” including nutrient-driven immune reprogramming that sustains pro-inflammatory macrophage states [67]. Cytokines such as IL-1β, IL-6, and IL-18 contribute to mesangial expansion, podocyte loss, and glomerulosclerosis [68].

In this setting, IL-4 is studied as a counter-regulatory signal, helping to modulate metabolic inflammation in DN. Gu et al. [44] reported in *db/db* mice that infusion of IL-4–polarized M2 macrophages shifted the renal immune environment toward an anti-inflammatory profile and reduced fibrosis. Mechanistically, the authors linked this effect to suppression of JAK2/STAT3 signaling in mesangial cells, suggesting cross-talk between IL-4–programmed macrophages and pro-inflammatory JAK2/STAT3-driven pathways.

#### Other chronic nephropathies

Outside LN and DN, IL-4–associated effects vary across disease contexts. In membranous nephropathy (MN) and focal segmental glomerulosclerosis (FSGS), higher IL-4/IL-13 signatures have been associated with treatment response. For example, cyclophosphamide treatment in MN has been reported to promote M2 polarization and increase IL-4/IL-13 levels via miR-223 upregulation [47]. In MN patients, successful immunosuppression has been associated with increased serum IL-4 and an inverse relationship with disease severity [69]. In FSGS, a Th2-predominant profile (high IL-4/IgE) has been reported to identify patients with better steroid responsiveness than those with Th17-skewed profiles [70].

Conversely, IL-4 signaling has been associated with more severe pathology in other settings. In IgA nephropathy (IgAN), elevated serum IL-4 and JAK–STAT6 activation have been linked to severe disease, whereas corticosteroid therapy has been associated with suppression of this axis and clinical stabilization [71]. Under hemodynamic stress, IL-4 may promote fibrosis: Cavalcante et al. [46] reported that IL-4 primed macrophages to produce TGF-β1 and YM1 in hypertensive mice, contributing to interstitial fibrosis, and clinical data linked higher IL-4 and reduced Treg signatures with endothelial injury in malignant hypertension [72]. Sustained local IL-4 signaling may also contribute to relapse and chronicity; elevated urinary IL-4 has been associated with frequent relapses in minimal change disease (MCD) and with glomerular sclerosis in FSGS [73]. Mechanistically, B cell–derived IL-4 has been reported to induce podocyte foot process effacement via STAT6 independent of immune complexes [45]. Thus, systemic IL-4 signatures may track with steroid sensitivity in some settings, whereas persistent local IL-4 activation may directly exacerbate renal injury.

The complexity of human CKD presents a significant barrier to translation. Conflicting clinical data, where IL-4 appears protective in some cohorts and pathogenic in others, likely reflect diverse patient profiles that simplified animal models fail to capture. Moreover, the reliance on the M1/M2 paradigm is increasingly problematic, as it fails to encompass the true heterogeneity of renal myeloid cells. As discussed further below, more nuanced models are needed to guide effective therapeutic strategies.

## Critical perspectives: beyond the M1/M2 dichotomy

The M1/M2 macrophage paradigm has been widely used to classify immune responses in renal injury, but it increasingly appears as an oversimplification of the complex in vivo reality. Wen et al. [74] noted that phenotypes induced in vitro often do not fully replicate the dynamic in vivo conditions. Macrophages during renal injury frequently exhibit mixed phenotypes, expressing both M1 and M2 markers simultaneously. Furthermore, Wen et al. highlighted that functional heterogeneity in macrophages is not solely defined by polarization signals but is also influenced by their origin, particularly the distinction between kidney-resident macrophages and infiltrating monocytes. This challenges the binary M1/M2 framework and calls for a more nuanced understanding of macrophage biology in renal disease.

Recent advances in single-cell technologies, as reviewed by Bell and Denby [75], have provided further insights into the complexity of renal myeloid cell populations. Conway et al. [76] used scRNA-seq to map myeloid cell subsets in murine renal fibrosis, identifying 12 distinct populations that did not fit conventional M1/M2 classifications. Notably, they found a subset of *Arg1* + monocytes co-expressing profibrotic markers such as *Tgfb1*, and another unique *Mmp12* + macrophage subset associated with the resolution phase. Building on these findings, Zhang et al. [77] combined scRNA-seq with spatial transcriptomics and identified a new ECM remodeling-associated macrophage (EAM) subset, which expresses high levels of fibrotic mediators and is spatially localized with fibroblasts. These EAMs promote fibrosis via the *Igf1-Igf1r* signaling axis.

Taken together, these findings suggest that future therapeutic strategies should move beyond broad M1/M2 targeting. Instead, interventions should selectively target specific pathogenic macrophage subsets based on their unique molecular signatures, rather than broadly suppressing macrophages essential for tissue homeostasis.

## Therapeutic interventions and translational challenges

**Fig. 3:**
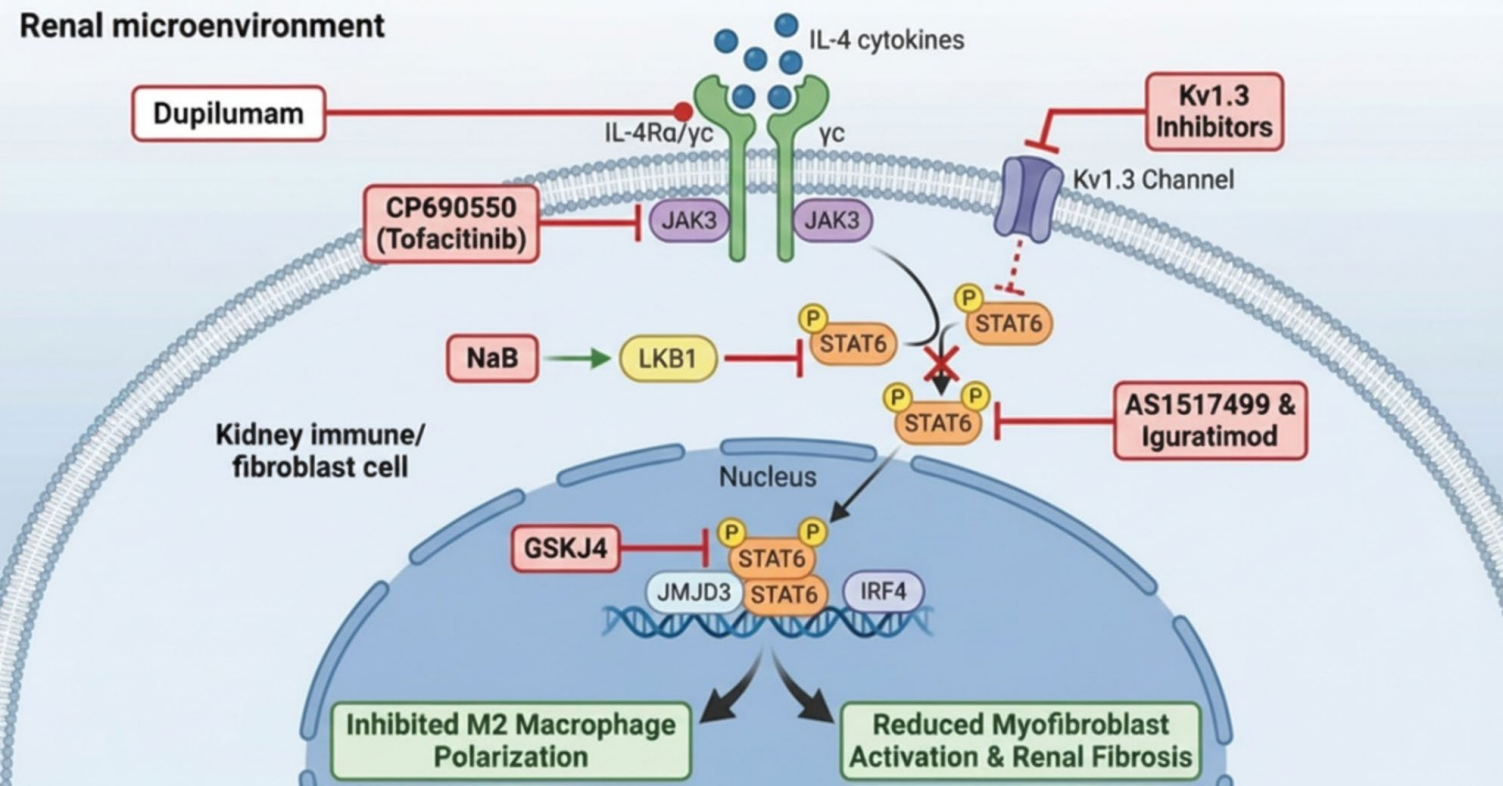
Multilevel Pharmacological Targeting of the IL-4/STAT6 Axis. Interventions target four mechanistic tiers: (1) Canonical Signaling: Direct blockade of the IL-4Rα/JAK3/STAT6 cascade (Dupilumab, Tofacitinib); (2) Epigenetic Control: Inhibition of the JMJD3-mediated *Irf4* switch; (3) Bioelectric-Metabolic Modulation: Kv1.3 channel blockade and NaB-driven LKB1 restoration; and (4) Pleiotropic Intervention: Dual suppression of STAT6 and SRC signaling by Iguratimod. Red inhibitory bars denote checkpoints arresting M2 polarization and ECM deposition

IL-4 has a dual role in kidney disease: it can support acute repair yet also contribute to chronic fibrogenesis. This biology complicates drug development. Although multiple approaches have shown benefit in preclinical models, consistent clinical translation has been limited. Because IL-4 can be protective in the post-injury phase, non-selective inhibition may impair tubular repair and/or perturb systemic immunity. Here, we summarize candidate interventions and discuss their mechanistic rationale alongside the safety and feasibility issues that currently constrain clinical application (Fig. 3; Table 2).

### JAK-STAT blockade: translational opportunities and risks

Targeting the canonical JAK–STAT pathway is a primary strategy for countering IL-4-driven fibrosis. Dupilumab, a monoclonal antibody that targets IL-4Rα, is a strong candidate for repurposing in kidney disease. By binding to IL-4Rα, Dupilumab blocks IL-4R and IL-13 signaling, preventing receptor heterodimerization and JAK activation [26]. Its proven efficacy and safety in Th2-mediated diseases, such as severe asthma and atopic dermatitis, suggest its potential to block renal scarring [78]. However, a key concern in the renal context is that inhibiting IL-4 may interfere with the “repair phase” of AKI, where IL-4 signaling plays a crucial role in tissue regeneration. This risk, which distinguishes renal applications from those in atopic conditions, underscores the need for precise temporal stratification during clinical translation.

In addition to receptor blockade, intracellular kinase inhibitors offer an alternative approach. The JAK3 inhibitor Tofacitinib (CP690,550) inhibits STAT6 phosphorylation in monocytes, reducing myofibroblast accumulation in murine models of obstructive nephropathy [79]. However, Tofacitinib’s clinical use is limited by significant toxicity, including venous thromboembolism (VTE), major adverse cardiovascular events (MACE), and malignancies, as identified in post-marketing surveillance. These systemic risks present a major obstacle to its use in CKD patients, who are already at high risk for cardiovascular and immune-related comorbidities [80].

Targeting the transcription factor STAT6 itself offers a strategy to bypass upstream toxicities, providing a more targeted therapeutic option. The selective STAT6 inhibitor AS1517499 has been shown to reduce M2 macrophage polarization in renal interstitial cells, offering promising results in preclinical models [81]. Despite this potential, the development of selective inhibitors for “undruggable” transcription factors remains a significant challenge, as these targets lack well-defined binding sites for small molecules [82].

### Targeting JMJD3: from chemical probes to therapeutics

Unlike broad kinase blockade, intervening at epigenetic effectors may separate pathological fibrogenesis from essential homeostatic signaling. In the IL-4 pathway, STAT6 activation induces JMJD3 (KDM6B), which removes the repressive H3K27me3 mark at the *Irf4* promoter. This step is required for chromatin opening and for initiation of the MMT program. In murine models, the JMJD3 inhibitor GSK-J4 attenuates fibrosis [83]. However, GSK-J4 is best regarded as a tool compound rather than a clinical candidate. As an ester prodrug, it is rapidly hydrolyzed in vivo to GSK-J1, which retains enzymatic activity but is poorly cell-permeable [84]. Together with limited metabolic stability and ongoing concerns about target selectivity [85], these properties currently preclude systemic therapeutic use. Translation will therefore require either improved JMJD3 inhibitors with drug-like pharmacokinetics or kidney-targeted delivery strategies that reduce systemic exposure while preserving local efficacy.

### Emerging therapeutic modalities

Modulating cellular bioelectricity provides an alternative route to disrupt IL-4-STAT6 signaling. The voltage-gated potassium channel Kv1.3 regulates Ca²⁺ entry that is necessary for STAT6 phosphorylation, thereby linking immune activation to tissue remodeling [40]. Kv1.3 blockade—using small molecules (e.g., PAPTP, Psora-4) or peptide inhibitors (e.g., dalazatide, HsTX1[R14A])—depolarizes the membrane and can dampen the IL-4 axis, limiting macrophage-driven fibrotic responses [86]. Clinical translation, however, faces modality-specific barriers. Many small-molecule inhibitors show cross-reactivity within the Kv family because of conserved channel architecture, raising concerns about off-target effects, including cardiac toxicity. Peptide therapeutics, in contrast, must address proteolytic degradation and potential immunogenicity. Given the widespread distribution of Kv1.3, progress in CKD will likely depend on highly selective agents (such as HsTX1[R14A]) and kidney-targeted delivery to minimize systemic exposure [87].

In parallel, metabolic reprogramming with sodium butyrate (NaB) has been proposed to act through the LKB1-STAT6 inhibitory axis. As a histone deacetylase inhibitor, NaB increases LKB1 (STK11), liver kinase B1 expression and suppresses IL-4-induced STAT6 phosphorylation. This mechanism is supported by *loss-of-function* data showing that LKB1 knockdown abrogates the ability of NaB to block M2 polarization and renal fibrosis [41]. Translation is nevertheless complicated by pharmacological pleiotropy: NaB functions as a broad histone deacetylase (HDAC) inhibitor and also activates G protein–coupled receptor (GPCR) signaling, making renal specificity difficult to achieve. Its rapid clearance further limits sustained exposure. Strategies such as kidney-targeted delivery or more stable analogues may be needed to concentrate activity in the kidney while reducing systemic effects [88].

Finally, iguratimod is used clinically for rheumatoid arthritis (RA) and has been reported to inhibit STAT6 and SRC signaling [39]. However, its direct molecular targets in renal cells have not been defined, which complicates mechanistic interpretation and risk assessment. Further validation in human kidney tissue and clinically relevant models will be required to clarify its mode of action and potential off-target liabilities.

### Overarching translational barriers

Overall, the approaches discussed in Sects.  “JAK-STAT blockade: translational opportunities and risks”–“Emerging therapeutic modalities” indicate that the main obstacles are not the lack of plausible targets, but four recurring translational barriers.

Current interventions do not adequately account for the dual role of IL-4 across injury and repair. As suggested by studies using receptor blockade, non-selective suppression may blunt the early reparative phase after AKI. Therefore, translation will require more than inhibiting the pathway: it will require defining when IL-4-STAT6 signaling supports adaptive repair and when it instead promotes maladaptive fibrogenesis.

Systemic inhibition that is pharmacologically effective may be poorly tolerated in CKD. For example, cardiovascular liabilities reported with some JAK inhibitors and the broad, multi-pathway effects of HDAC inhibitors are particularly problematic in patients with substantial vascular and metabolic comorbidity. This vulnerability lowers the margin for adverse events (including VTE), making long-term, broad systemic exposure difficult to justify.

Across modalities, insufficient kidney exposure remains a common reason for failure. Rapid in vivo conversion of epigenetic probes (e.g., GSK-J4 to the poorly permeable GSK-J1) and proteolytic instability of peptide inhibitors illustrate how validated targets can remain difficult to engage in vivo. Progress will likely depend less on further affinity gains and more on delivery solutions—especially kidney-targeted or kidney-retentive strategies that achieve local target engagement while limiting systemic exposure.

Differences between rodent and human immunity also limit predictability. Murine models may not capture key features of human macrophage states or the cytokine environments typical of clinical CKD. Consequently, findings from mice should be confirmed in human**-**relevant systems (e.g., primary human cells, kidney organoids, or ex vivo tissue) before clinical translation.

**Table 2:** Therapeutic Interventions Targeting the IL-4/STAT6 Axis and Translational Barriers

Therapeutic Strategy	Representative Agent / Target	Mechanism	Therapeutic Advantages	Critical Limitations & Challenges
Receptor Blockade	Dupilumab [Clinical] (Target: IL-4Rα) [78]	Sterically blocks receptor dimerization; halts JAK signaling.	Drug Repurposing: Proven safety in asthma/atopic dermatitis; dual blockade of IL-4/IL-13.	Interference with Repair: Risk of inhibiting the requisite “repair phase” in AKI/tubular regeneration.
Kinase Inhibition	Tofacitinib [Clinical] (Target: JAK3) [79]	Inhibits intracellular phosphorylation cascade; reduces myofibroblast accumulation.	Potency: Robust suppression of downstream STAT6 signaling.	Systemic Toxicity: Linked to VTE, MACE, and malignancies; high risk for comorbid CKD patients.
Transcription Factor Targeting	AS1517499 [Preclinical] (Target: STAT6) [81]	Selectively suppresses transcriptional activity of STAT6.	Specificity: Directly targets the terminal driver of M2 polarization.	“Undruggable” Nature: Lack of defined binding pockets hinders pharmacological development.
Epigenetic Reprogramming	GSK-J4 [Preclinical] (Target: JMJD3) [83]	Prevents H3K27me3 demethylation at *Irf4* promoter; blocks MMT.	Lineage Control: Specifically blocks lineage plasticity without compromising cell viability.	pharmacokinetic instability: Rapid hydrolysis to cell-impermeable metabolite (GSK-J1); serves only as a chemical probe.
Bioelectric Modulation	Small mols (PAPTP, Psora-4); Peptides (Dalazatide, HsTX1[R14A]) [Preclinical] (Target: Kv1.3) [40, 86]	Gates Ca²⁺ influx required for STAT6 phosphorylation.	Novel Vantage Point: Uncouples immune activation via biophysical properties.	Modality Hurdles: Off-target cardiac toxicity (small molecules) or proteolytic instability (peptides).
Metabolic Reprogramming	NaB[Preclinical] (Target: HDAC/LKB1) [41]	HDAC inhibition upregulates LKB1, suppressing STAT6 phosphorylation.	Endogenous Re-activation: Leverages an intrinsic inhibitory axis (LKB1) to counteract IL-4 signaling.	Promiscuous Pharmacology: Pan-HDAC inhibition and GPCR agonism cause systemic pleiotropy.
Pleiotropic Intervention	Iguratimod [Clinical] (Target: STAT6/SRC/Unknown) [39]	Dual inhibition of STAT6 and SRC signaling.	Synergistic Efficacy: “One drug, multiple targets”; established safety in RA.	Target Uncertainty: Undefined renal molecular targets warrant rigorous validation.

Abbreviations: AKI, acute kidney injury; CKD, chronic kidney disease; IL-4, interleukin-4; IL-13, interleukin-13; IL-4Rα, interleukin-4 receptor alpha; JAK, Janus kinase; JAK3, Janus kinase 3; STAT6, signal transducer and activator of transcription 6; M2, alternatively activated (type 2) macrophage polarization; JMJD3 (KDM6B), Jumonji domain–containing protein 3 (lysine demethylase 6B); H3K27me3, trimethylation of histone H3 lysine 27; IRF4, interferon regulatory factor 4; MMT, macrophage-to-myofibroblast transition; Kv1.3, voltage-gated potassium channel Kv1.3 (KCNA3); Ca²⁺, calcium ion; NaB, sodium butyrate; HDAC, histone deacetylase; LKB1 (STK11), liver kinase B1 (serine/threonine kinase 11); GPCR, G protein–coupled receptor; SRC/Src, SRC proto-oncogene, non-receptor tyrosine kinase; VTE, venous thromboembolism; MACE, major adverse cardiovascular events; RA, rheumatoid arthritis

## Conclusions

Overall, available evidence suggests that IL-4 acts in two directions in kidney disease: it supports recovery after acute, reversible injury, but during persistent stress it may promote fibrogenesis by reprogramming myeloid cells through IL-4/STAT6 signaling, including the STAT6–JMJD3–IRF4 axis and MMT. The inconsistent associations reported across human CKD cohorts are likely driven by differences in disease stage, tissue compartment, and immune milieu, which argues for precision strategies rather than non-selective pathway blockade.

The field has also moved beyond the binary “M1/M2” framework. Single-cell and spatial studies instead describe diverse myeloid states, including subsets such as EAMs, shaped by local niche signals and metabolic constraints. A practical implication is that therapeutic development should focus on context-specific targets (e.g., JMJD3, Kv1.3) and delivery strategies that preferentially modulate profibrotic programs while preserving protective immune functions.

## Data Availability

No datasets were generated or analysed during the current study.
